# Comparison of the detection performance of two different one-step-combined test strips with fluorescent microspheres or colored microspheres as tracers for influenza A and B viruses

**DOI:** 10.1186/s12985-019-1190-0

**Published:** 2019-07-19

**Authors:** Qingjun Pan, Weiquan Wu, Shuzhen Liao, Sijie Wang, Chunfei Zhao, Chen Li, Ping Wu

**Affiliations:** 1 Institute of Nephrology, Affiliated Hospital of Guangdong Medical University, Zhanjiang, Guangdong China; 2 Clinical Research Center, Affiliated Hospital of Guangdong Medical University, Zhanjiang, Guangdong China; 3 Department of Clinical Laboratory, Affiliated Hospital of Guangdong Medical University, Zhanjiang, Guangdong China

**Keywords:** Influenza A, Influenza B, Fluorescent microspheres, Colored microspheres, One step combined test

## Abstract

**Background:**

Influenza A and B viruses mainly cause respiratory infectious disease. Till now, few tests are able to simultaneously detect both, especially in primary medical establishments.

**Methods:**

This study was designed to compare the performance of two different one-step-combined test strips for the detection of influenza A and B: one strip with fluorescent microspheres for tracers (FMT); and the other strip with colored microspheres for tracers (CMT). To test the strips, cultures of influenza A, B, and other pathogenic viruses were used, in addition to 1085 clinical specimens from symptomatic patients with respiratory infections. Real-time RT-PCR was also considered as a reference method used to detect the different results of FMT and CTM.

**Results:**

Detection thresholds for influenza A and B cultures using serial dilutions revealed that the sensitivity of FMT was higher than that of CMT (both *P* < 0.05). With the culture mixtures of Coxsackie virus (A16), enteric cytopathic human orphan virus (ECHO type30), enterovirus (EV71), rotavirus (LLR strain), and enteric adenovirus (AdV 41), specificity assessment demonstrated that there was no cross reaction during the usage of the two test strips as shown by the results which were negative. In the detection of influenza A in 1085 clinical specimens, the total coincidence rate was 96.7%, the positive coincidence rate was 97.1%, and the negative coincidence rate was 96.7%. In the case of influenza B detection, the total coincidence rate was 99.1%, the positive coincidence rate was 92.6%, and the negative coincidence rate was 98.5%. In addition, with influenza A or B real-time RT-PCR detection method, the results showed that, for influenza A, 26 of the 33 specimens that negative with CMT but positive with FMT, showed positive results, and none of the 3 specimens that positive with CMT but negative with FMT showed a positive result; For influenza B, 12 of the 15 specimens that negative with CMT but positive with FMT, showed positive results, and none of the 5 specimens that positive with CMT but negative with FMT showed a positive result.

**Conclusions:**

FMT performed better than CMT in the combined detection of influenza A and B viruses.

## Background

Influenza is caused by a type of virus that mainly attacks the upper respiratory tract (nose and throat). It can also affect parts of the lower respiratory tract such as bronchi and rarely lungs [1]. There are three types of influenza viruses: A, B, and C. Influenza A and B are the two types that routinely spread in humans and cause seasonal flu epidemics with severe symptoms. Symptoms of type C flu are much less severe. Influenza A and B viruses infect 5 to 15% of the global population annually and cause an estimated 250,000 to 500,000 deaths [2]. Outbreaks of influenza regularly cause excess mortality among the elderly and considerable morbidity in all the age groups during the influenza season [3, 4]. Therefore, influenza A and B viruses are tested for simultaneously during the clinical diagnosis of viral respiratory infections.

For the identification of the type of virus causing the influenza infections, several methods, such as immunological and molecular biological methods are used [5, 6]. Immunological methods currently available are the ones that directly detect the presence of IgM and IgG antibodies in the specimens [7, 8]. Most of these methods are limited with respect to technology or equipment, especially in primary medical institutions and field tests.

The use of fluorescent microspheres for tracers is an emerging technique in antigen–antibody reactions. They are based on immunochromatographic assays and have been used widely in several fields, such as medical science, food security and so on [9, 10]. Compared to colloidal gold detection [11], the use of fluorescent microspheres for tracers is rapid, sensitive, and reliable [12–14]. Following lighting with an ordinary UV lamp or special equipment, reaction bands of the fluorescent microspheres for tracers can be observed with the naked eye. After the development of fluorescent microspheres detection technique, certain relevant single-item detection products based on it have been developed [15–17]. Colored microspheres for tracer detection are also used in antigen–antibody reactions. They can be produced in any color and from materials ranging in size from 10 μm to 1000 μm, such as silica, polythene, and so on [18, 19]. However, there is insufficient data on the sensitivity of colored microspheres for tracers, which will be assessed in the current study.

The objective of the current study was to compare the fluorescent microspheretracers (FMT) with colored microspheretracers (CMT), and to discuss the efficiency of FMT in relation to CMT in combined influenza A + B detection. This will lead to a credible method for detecting influenza A and B infections and will benefit the clinical diagnostic and therapeutic fields, especially the primary medical establishments.

## Materials and methods

### Viruses

Viruses used included influenza A (A/PR/8/34) and B (B/Guangzhou/01/2007), rotavirus (LLR strain), enteric adenovirus (AdV type 41), Coxsackie virus (A16), enteric cytopathic human orphan virus (ECHO) 116 (type 30), and enterovirus (EV71) were purchased from American Type Culture Collection (ATCC) or provided by Guangzhou Women and Children Medical Center. They were multiplied in cell cultures with Madin-Darby canine kidney (MDCK) cells, MA-104 cells, Hela cells, Hep-2 cells, rhabdomyosarcoma (RD) cells, and Vero cells which were bought from ATCC using Dulbecco’s modified Eagle’s medium (DMEM) (Gibco) or Rosewell Park Memorial Institute (RPMI) (Gibco) culture medium with 10% fetal bovine serum (FBS) (HyClone), and separately stored in the laboratory.

### Clinical samples

Clinical specimens including both shallow nasal and NP swabs were collected from 1085 symptomatic patients with respiratory infections from the in- and out-patient wards at the Affiliated Hospital of Guangdong Medical University, and from the Guangzhou Women and Children Medical Center between March 2015 and February 2017. The study participants included 672 males and 413 females of the mean age 14.7 ± 18.3 years. All the patients supplied written informed consent to participate. The study was approved by the ethics committee of the Affiliated Hospital of Guangdong Medical University (LL201403186259).

During and after the data collection, all the authors had obtained the information that could identify individual participants.

### Two different one-step-combined test strips for influenza a and B

**Fig. 1:**
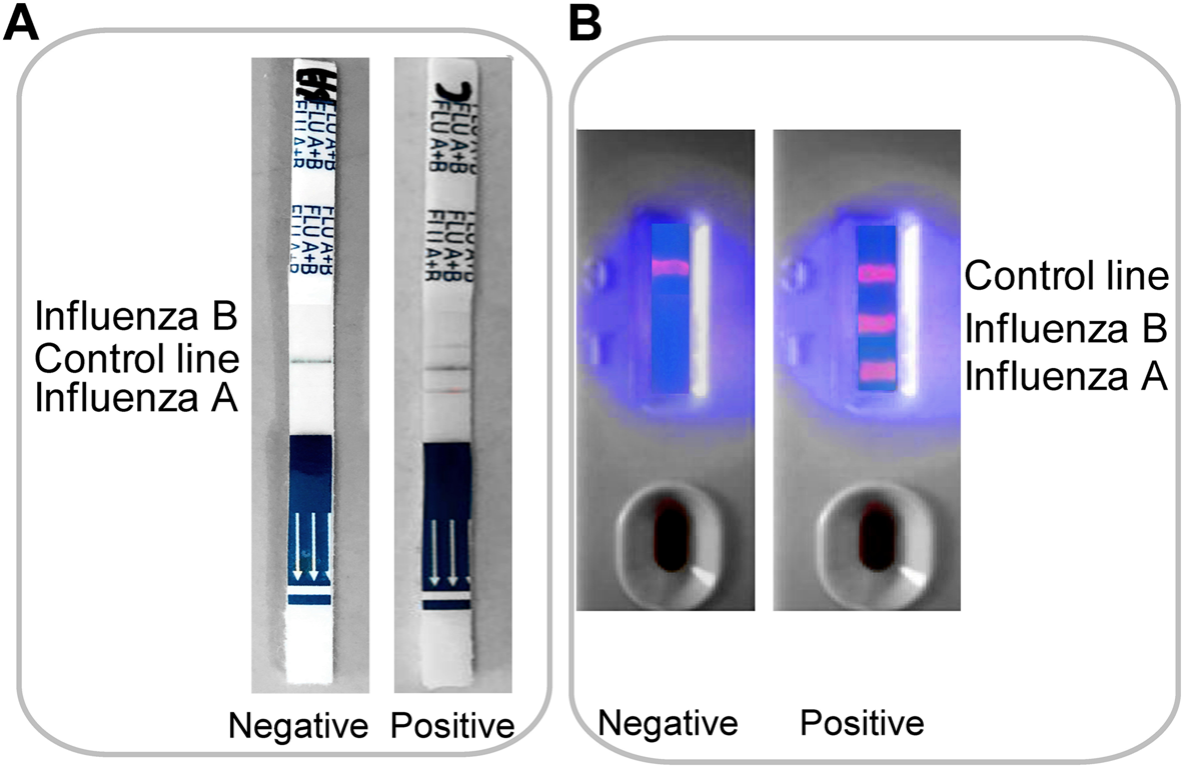
Test modes of the two combined detection test strips**. a** Colored microspheres for tracers (CMT): Control line (blue); Detection line (red) including the influenza A line (near the sample loading end) and the influenza B line. Interpretation of results: after the test procedure, negative (left) and double positive (right) results were detected. **b** Fluorescent microspheres for tracers (FMT): Control line; Detection line including the influenza A line (near the sample loading well) and the influenza B line. Interpretation of results: after the test procedure, negative (left) and double positive (right) results were detected

The combined influenza A + B fluorescent microspheres test strip (FMT) was supplied by Guangzhou Yuemo Biotechnology Co., Ltd. (Guangzhou, China). The tracers were fluorescent microspheres (phenylethene luminative monomeric copolymers; approximate diameter 200 nm) with excitation and emission wavelengths of 360 nm and 615 nm, respectively (Suzhou Vdo Biotech Co., Ltd., Suzhou, China). The detection procedure was performed according to the manufacturer’s instructions, and the detection mode is shown in Fig. 1a.

A CerTest influenza A + B blister test strip (CMT) was supplied by CerTest Biotec S.L. (Zaragoza, Spain) for the qualitative detection of influenza type A and type B from nasal swab, nasopharyngeal wash and aspirate specimens. The tracer is a colored microsphere. The detection procedure was performed according to the manufacturer’s instructions, and the detection mode is shown in Fig. 1b.

### Influenza a and B real-time reverse transcription polymerase chain reaction (real-time RT-PCR) kits

Influenza A and B real-time RT-PCR detection kits were purchased from Guangdong Huayin Pharmaceutical Technology Co., Ltd. (Guangzhou, China). The kits contain a specific ready-to-use system for the detection of the Influenza A or B by Reverse Transcription Polymerase Chain Reaction (RT-PCR) in the real-time PCR system. The master contains a Super Mix for the specific amplification of Influenza A or B virus RNA. The reaction is done in one step real time RT-PCR. The first step is a reverse transcription (RT), during which the Influenza A or B RNA is transcribed into cDNA. Afterwards, a thermostable DNA polymerase is used to amplify the specific gene fragments by means of polymerase chain reaction (PCR). Fluorescence is emitted and measured by the potical unit of the real time systems during PCR. An external positive control contained, allows the determination of the gene load. Followed, the testing process was according to the manufacturer’s instructions. The tested clinical specimens were same to one-step-combined test strips for influenza A and B.

### Sensitivity valuation

Influenza A and B were diluted two fold serial dilution and detected using the two strips. The initial concentrations of influenza A and B were 7.4 × 10^5^ plaque-forming units (PFU)/mL and 4.2 × 10^5^ PFU/mL, respectively. Three drops (150 μL each) of the viral culture mixture were synchronously applied to the sample loading well of the detection strips. The results were recorded within 10 min.

### Specificity valuation

For specificity assessment, cultures of Coxsackie (A16), ECHO (type 30), entero- (EV71), rota (LLR strain), and enteric adeno (AdV 41) viruses were used.

### Preparation of clinical samples

Clean bamboo sticks were used to effectively mix and liquefy the fresh specimens comprising mainly mucus, pus, and blood. A thin tube was used to aspirate three drops (150 μL each) of the mixture, which was synchronously applied to each of the detection cards.

### Statistical analyses

The total coincidence rate, positive coincidence rate, and the negative coincidence rate of CMT and FMT were calculated to assess detection in the clinical specimens [20]. Statistical analysis was performed using a Student’s t test. *P* < 0.05 was considered statistically significant. Statistical analysis was performed with SPSS 15.0.

The definition and way of calculation of total coincidence rate, positive coincidence rate, and negative coincidence rate as followed: Total coincidence rate = 100% × [(FMT&CMT both positive+ FMT&CMT both negative)/total samples]; Compared to CMT, the positive coincidence rate of FMT = 100% × [FMT&CMT both positive /(FMT&CMT both positive + CMT positive but FMT negative)], and the negative coincidence rate of FMT = 100% × [FMT&CMT both negative /(FMT&CMT both negative + CMT negative but FMT positive)].

## Results

### Sensitivity valuation of CMT and FMT

After the two-fold serial dilutions of the influenza A cultures to 1/2^8^ (58 × 10^2^ PFU/mL), the CMT results were negative, whereas viruses were still detected as positive result with FMT, till the dilutions of 1/2^9^ (29 × 10^2^ PFU/mL) as negative. After the two-fold serial dilutions of the influenza B cultures to 1/2^7^ (16.4 × 10^2^ PFU/mL), the CMT results were negative, whereas viruses were still detected as positive result with FMT, till the dilutions of 1/2^8^ (8.2 × 10^2^ PFU/mL) as negative. Statistical analysis revealed the sensitivity of FMT was significantly higher than that of CMT (both *P* < 0.05) (Table 1).

**Table 1:** Comparison of the results of the two test strips at two-fold serial dilutions of influenza A and B cell cultured samples

Groups Methods		Two-fold serial dilutions									
		1/2	1/2^2^	1/2^3^	1/2^4^	1/2^5^	1/2^6^	1/2^7^	1/2^8^	1/2^9^	1/2^10^
Influenza A	CMT	+	+	+	+	+	+	+	–	–	–
	FMT	+	+	+	+	+	+	+	+	+	–
Influenza B	CMT	+	+	+	+	+	+	–	–	–	–
	FMT	+	+	+	+	+	+	+	+	–	–

### Specificity valuation of CMT and FMT

The detection results from the two test strips (Table 2) revealed that the culture mixture comprising Coxsackie (A16) (5.2 × 10^6^ TCID_50_/mL), ECHO (type30) (3.3 × 10^6^ TCID_50_/mL), entero- (EV71) (2.5 × 10^6^ TCID_50_/mL), rota (LLR strain) (3.7 lgCCID_50_/mL), and enteric adeno (AdV 41) viruses (4.8 × 10^4^ TCID_50_/mL) had no cross reaction, as shown by the test results which were negative.

**Table 2:** Comparison of the test results with other viruses

Methods	Coxsackie virus (A16)	ECHO virus (type 30)	Enterovirus (EV71)	Rotavirus (LLR strain)	Enteric adenovirus (AdV 41)
CMT	–	–	–	–	–
FMT	–	–	–	–	–

### Detection of influenza a and B in clinical specimens using CMT and FMT

Both strips were used to detect 1085 specimens, and the results are presented in Tables 3 and 4.

**Table 3:** Comparison of the test results of influenza A clinical specimens with the two test strips

FMT	CMT		Total
	Positive	Negative	
Positive	101	33	134
Negative	3	948	951
Total	104	981	1085

**Table 4:** Comparison of the test results of influenza B clinical specimens with the two test strips

FMT	CMT		Total
	Positive	Negative	
Positive	63	15	78
Negative	5	1012	1007
Total	58	1027	1085

For influenza A detection, the total coincidence rate of CMT and FMT was 96.7% [(101 + 948)/1085)]. Compared to CMT, the positive coincidence rate of FMT was 97.1% [101/(101 + 3)], and the negative coincidence rate of FMT was 96.7% [948/(948 + 33)]. For influenza B detection, the total coincidence rate of CMT and FMT was 99.1% [(63 + 1012)/1085)]. Compared to CMT, the positive coincidence rate of FMT was 92.6% [63/(63 + 5)], and the negative coincidence rate of FMT was 98.5% [1012/(1012 + 15)].

### Detection of the different results of clinical specimens by CMT and FMT using real-time RT-PCR

For the different results of clinical specimens by CMT and FMT, an influenza A real-time RT-PCR detection kit was also used to detect influenza A again [21]. The results revealed that 26 of the 33 specimens that detected negative with CMT but positive with FMT, detected positive with the real-time RT-PCR detection method, and none of the 3 specimens that had previously detected positive with CMT but negative with FMT provided a positive result (Table 5).

**Table 5:** Detection of the different results of clinical specimens by CMT and FMT using influenza A real-time RT-PCR

RT-PCR	Influenza A		Total
	CMT^−^FMT^+^	CMT^+^FMT^−^	
Positive	26	0	26
Negative	7	3	10
Total	33	3	36

Also, for the different results of clinical specimens by CMT and FMT, an influenza B real-time RT-PCR detection kit was also used to detect influenza B again [22]. The results revealed that of the 15 specimens that had previously detected negative with CMT but were positive with FMT, 12 detected positive with real-time RT-PCR, and none of the 5 specimens that had previously detected positive with CMT but negative with FMT detected positive with real-time RT-PCR (Table 6).

**Table 6:** Detection of the different results of clinical specimens by CMT and FMT using influenza B real-time RT-PCR

RT-PCR	Influenza B		Total
	CMT^−^FMT^+^	CMT^+^FMT^−^	
Positive	12	0	12
Negative	3	5	8
Total	15	5	20

## Discussion

There is a gradual increase in the global usage of colloidal gold detection of influenza A and B in samples. However, these are rare combined detection products and possess technological limitations. In scientific research and clinical practice, the CerTest influenza A + B one step card test (CMT) has been welcomed [23]. However, during clinical detection, sometimes CMT strips fail to detect viruses and provide false negative results due to the shortage of colored microspheres for the tracer detection technique, especially in cases where the quantities of influenza A and B specimens are insufficient. Therefore, there is an enormous requirement for highly sensitive fluorescent microspheres as tracers based on immunochromatographic assays. At present, there are limited manufacturers of combined influenza A + B fluorescent microsphere detection test strips all over the world.

In the current study, to assess the detection performance of combined influenza A + B fluorescent microsphere detection test strips, CMT and real-time RT-PCR were used as references. Using colored microspheres with different colors as tracers, CMT can produce meaningful results when used as diagnostic reagents in vitro. It can be used to determine the efficiency of detection. Therefore, colored microspheres for tracers based on immunochromatographic assays have gradually attracted attention in the medical science and food security fields [24–26]. In addition, real-time RT-PCR was considered as a reference method used to detect the different results of FMT and CTM. The results of real-time RT-PCR showed that 78.8% (26/33) of the specimens that detected influenza A negative with CMT but positive with FMT were also positive, which can be speculated that most of these samples were positive, so the sensitive of FMT was significantly higher than CMT. However, compared to real-time RT-PCR, FMT still have 21.2% (7/33) negative results. As well known, the detective method of real-time RT-PCR has difference with FMT and CMT, especially for the detective targets, nucleic acid for real-time RT-PCR method and protein antigens for FMT. Here, this study was designed to compare the performance of two different one-step-combined test strips CMT and FMT for the detection of influenza A and B, and real-time RT-PCR was considered as a reference method but not as the “gold standard”. As well known, virus isolation in cell cultures has long served as the “gold standard” for virus detection; however this approach is often slow and requires considerable technical expertise.

Influenza A is the most common cause of severe acute respiratory infectious diseases in children [27], and the most common pathogens have been widely studied in China [28, 29]. In the current study, combined influenza A + B FMT strips were used to detect viruses in clinical specimens. The rate of positive influenza A and B detection in 1085 clinical specimens was 12.4% (134/1085) and 7.2% (78/1085) respectively. It is necessary that both influenza A and B viruses are detected accurately in clinical specimens from patients with respiratory infectious disease due to the high infection rates in China.

The results indicate that the combined influenza A + B FMT detection strips were more sensitive than the CMT strips. Also, the detective procedures and timing of the FMT and CMT strips are similar. Based on the FMT strips have not been large-scale commercial application, the cost cannot be compared now. However, the FMT technique has some limitations which must be considered, such as the risk of false positive results. Therefore, caution should be used, especially while handling weakly positive specimens [30]. Another constraint is that the numbers of the positive results of the clinical specimen is not big enough. Therefore, there is a necessity for further development by medical establishments, and for confirmation by large-scale clinical specimens. To afford rapid, sensitive, and reliable diagnosis in clinical settings, the detection results should be procured within half an hour of specimen collection.

This study described a straightforward head-to head comparison of CMT and FMT test strips, which may be of great value to researchers involved in clinical diagnosis.

## Conclusions

Both FMT and CMT were able to detect both influenza A and B viruses with high specificity, but FMT had significantly higher detection sensitivity than CMT.

## Data Availability

The datasets used and analyzed in the current study are available from the corresponding author upon reasonable request.

## Abbreviations

CMT: Colored microsphere as tracers
FMT: Fluorescent microsphere as tracers
PFU: Plaque forming unit
Real-Time RT-PCR: Real-Time Reverse Transcription-PCR
